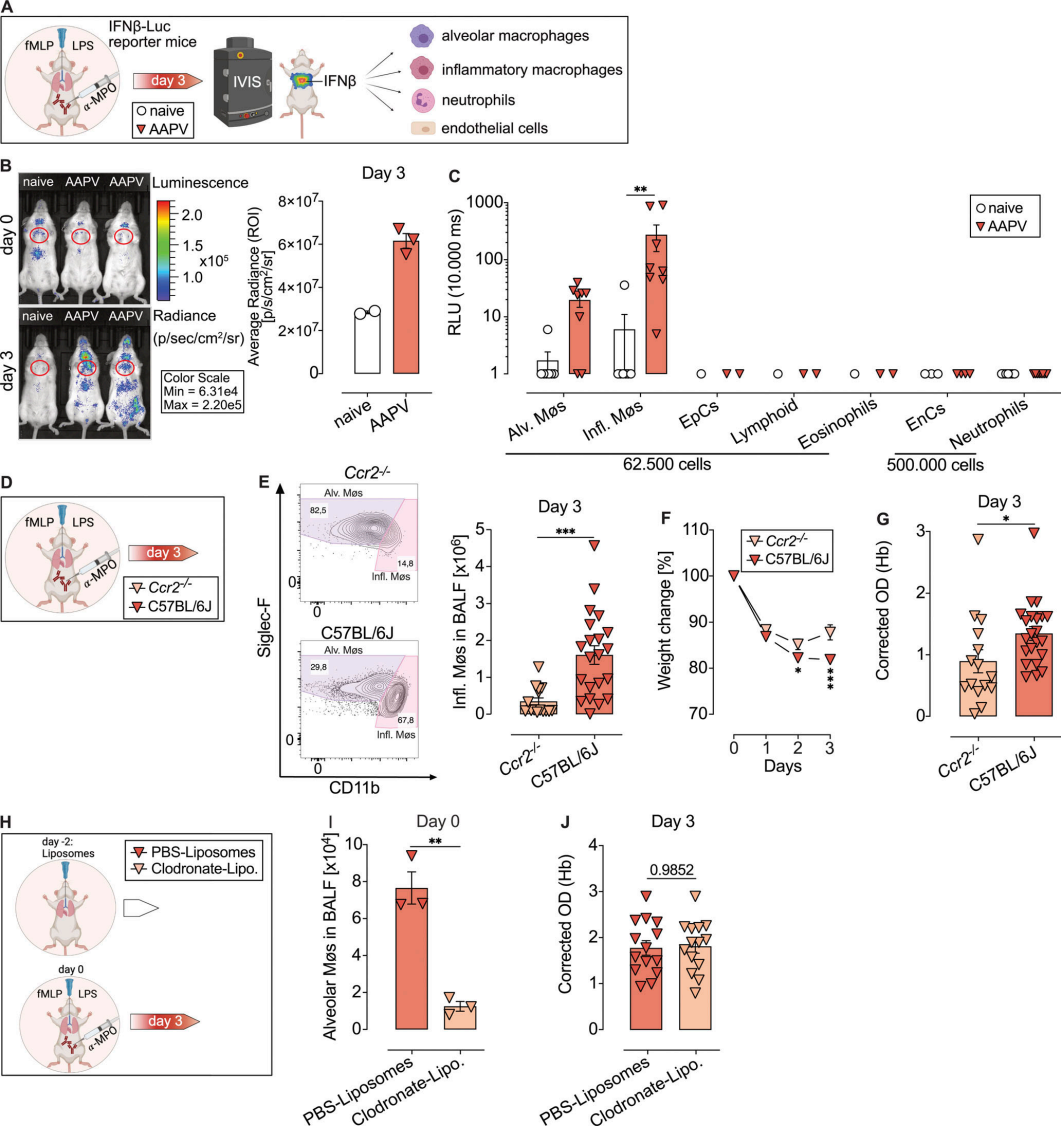# Correction: Monocyte-derived macrophages aggravate pulmonary vasculitis via cGAS/STING/IFN-mediated nucleic acid sensing

**DOI:** 10.1084/jem.2022075911022022c

**Published:** 2022-11-11

**Authors:** Nina Kessler, Susanne F. Viehmann, Calvin Krollmann, Karola Mai, Katharina M. Kirschner, Hella Luksch, Prasanti Kotagiri, Alexander M.C. Böhner, Dennis Huugen, Carina C. de Oliveira Mann, Simon Otten, Stefanie A.I. Weiss, Thomas Zillinger, Kristiyana Dobrikova, Dieter E. Jenne, Rayk Behrendt, Andrea Ablasser, Eva Bartok, Gunther Hartmann, Karl-Peter Hopfner, Paul A. Lyons, Peter Boor, Angela Rösen-Wolff, Lino L. Teichmann, Peter Heeringa, Christian Kurts, Natalio Garbi

Vol. 219, No. 10 | https://doi.org/10.1084/jem.20220759 | August 23, 2022

The authors regret that Figs. 1, 3, and 5 contained errors in the original version of their article. In Fig. 1 C, the color-coded diagnosis legend indicating MPO (dark yellow) or control (dark blue) was missing from the top of the figure panel. In Fig. 3 C, the color legend indicating DAPI (blue) and MPO (dark pink) staining was not visible. In Fig. 3 J, the Merge Zoom image for the *Sting*^*gt/gt*^ group was missing from the bottom right corner image. It should have shown a magnification of the bottom left image. In Fig. 5 B, the mouse legends “naive” and “AAPV” were missing. The corrected figures are shown here. These corrections do not alter the meaning of the figures or their conclusions, and the legends remain unchanged. The errors appear in print and in PDFs downloaded before October 31, 2022.

**Figure 1:**
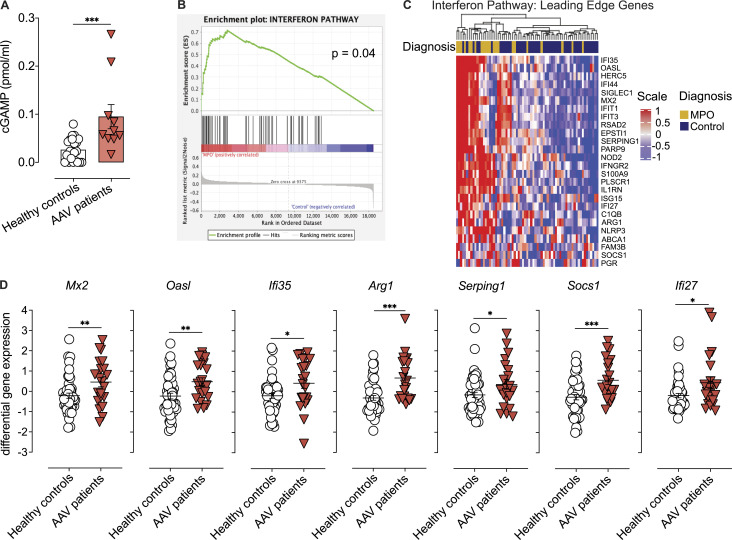


**Figure 3:**
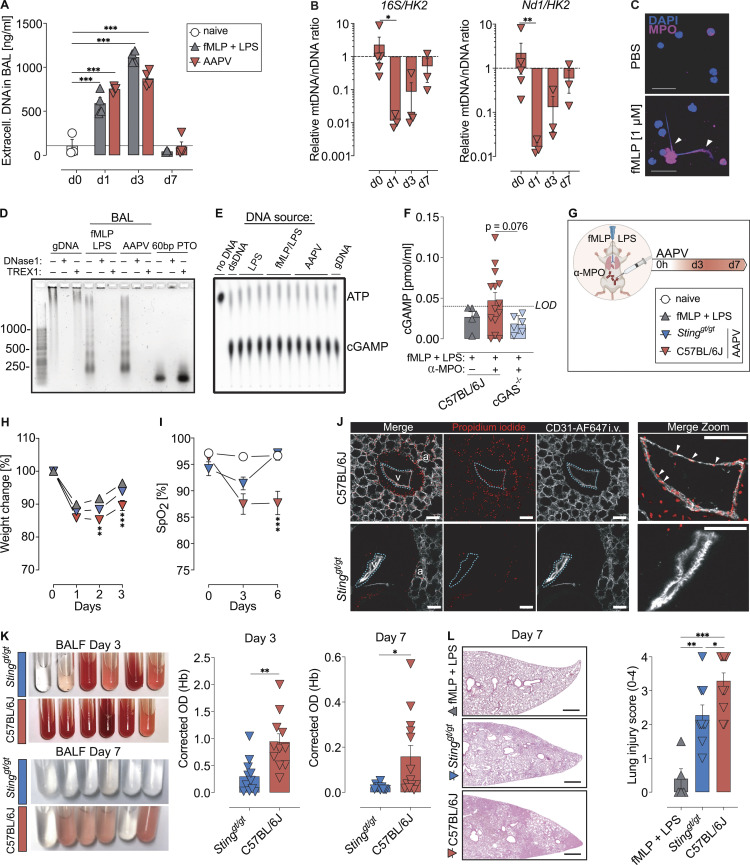


**Figure 5:**